# MR relaxation times of agar‐based tissue‐mimicking phantoms

**DOI:** 10.1002/acm2.13533

**Published:** 2022-04-12

**Authors:** Anastasia Antoniou, Leonidas Georgiou, Theodora Christodoulou, Natalie Panayiotou, Cleanthis Ioannides, Nikolaos Zamboglou, Christakis Damianou

**Affiliations:** ^1^ Department of Electrical Engineering Computer Engineering, and Informatics Cyprus University of Technology Limassol Cyprus; ^2^ Department of Interventional Radiology German Oncology Center Limassol Cyprus

**Keywords:** agar, MR relaxation times, MRgFUS, tissue‐mimicking phantoms

## Abstract

Agar gels were previously proven capable of accurately replicating the acoustical and thermal properties of real tissue and widely used for the construction of tissue‐mimicking phantoms (TMPs) for focused ultrasound (FUS) applications. Given the current popularity of magnetic resonance‐guided FUS (MRgFUS), we have investigated the MR relaxation times *T*1 and *T*2 of different mixtures of agar‐based phantoms. Nine TMPs were constructed containing agar as the gelling agent and various concentrations of silicon dioxide and evaporated milk. An agar‐based phantom doped with wood powder was also evaluated. A series of MR images were acquired in a 1.5 T scanner for *T*1 and *T*2 mapping. *T*2 was predominantly affected by varying agar concentrations. A trend toward decreasing *T*1 with an increasing concentration of evaporated milk was observed. The addition of silicon dioxide decreased both relaxation times of pure agar gels. The proposed phantoms have great potential for use with the continuously emerging MRgFUS technology. The MR relaxation times of several body tissues can be mimicked by adjusting the concentration of ingredients, thus enabling more accurate and realistic MRgFUS studies.

## 1 INTRODUCTION

Tissue‐mimicking phantoms (TMPs) are increasingly used for the preclinical validation of diagnostic and therapeutic modalities, reducing the use of animal subjects.^1^ Gel phantoms constitute a more economical and ergonomic solution for preclinical research compared to experimental animals, also given that their lifespan can be simply lengthened by adding preservatives.^1^, ^2^ Several categories of gelling agents, including agar,^3^ gelatin,^4^ polyacrylamide (PAA),^5^ poly‐vinyl alcohol,^6^ polyvinyl chloride,^7^ silicone,^8^ and TX‐151,^9^ have been used in the construction of gel phantoms for quality assessment purposes in medicine and biomedical research. Accurate replication of tissue properties is of great importance for the efficacy of such procedures, especially when evaluating therapeutic applications with clinical potential.

The current increasing application of focused ultrasound (FUS) in medicine^10^ requires the development of high‐quality TMPs specially designed for use with this specific technology to accelerate its clinical translation. The FUS‐induced thermal effects were proven to be essential in many oncological applications, thereby serving as an alternative therapeutic solution over surgical and systemic approaches.^11^ Thermal therapy with FUS is based on the ability to precisely focus extracorporeal ultrasonic waves into a millimeter‐sized area of malignant tissue, thus elevating the temperature to hyperthermic or ablative levels.^12^ Therefore, TMPs intended for FUS studies should be capable of accurately replicating both the acoustical and thermal characteristics of biological tissue. Under FUS exposure, the thermal behavior of a material is a function of various parameters, among which the most critical are the specific heat capacity, thermal conductivity, and thermal diffusivity.^13^, ^14^ Concerning acoustical characteristics, the key properties to be emulated are the speed of sound in the medium, acoustic impedance, and attenuation coefficient.^13^, ^14^

FUS treatment is typically applied under the US or magnetic resonance imaging (MRI) guidance,^11^ with MRI being the method of choice because of its superior imaging resolution and its ability to acquire temperature data by intraoperative MR thermometry.^15^, ^16^ The contrast in MR images emerges from changes in the proton density and the magnetic relaxation times *T*1 and *T*2 of tissues.^16^ Several animal studies have shown that the MR parameters of tissue greatly affect the contrast between normal untreated tissue and FUS‐ablated areas.^17^, ^18^ In fact, the MR relaxation times of FUS lesions were found to vary depending on the tissue type, suggesting that the MR properties of the host tissue define the MR appearance of lesions.^17^ More importantly, the temperature dependence of tissue relaxation times allows for noninvasive temperature monitoring during thermal applications.^16^, ^19^ Therefore, precise replication of MR relaxation parameters is essential for producing tissue‐like MR signals and more realistic temperature maps in the process of evaluating thermal protocols. It is thus of paramount importance that TMPs are both US and MR imageable and possess tissue‐like MR properties in order to be qualified for use with the magnetic resonance‐guided FUS (MRgFUS) technology.

So far, PAA, gelatin, and agar‐based phantoms were proven efficient to properly mimic biological tissues in thermal studies by replicating critical acoustical, thermal, and MR properties.^2^, ^3^, ^4^, ^5^ Agar and PAA gels are favorable in that they possess melting points sufficiently high for ablative FUS, whereas gelatin phantoms are only proposed for hyperthermia applications since they lack the capacity to withstand ablative temperatures.^2^

PAA gels are beneficial over agar gels in that they are transparent, allowing for visually discriminating coagulative areas.^5^, ^20^ These phantoms normally contain heat‐sensitive materials, such as bovine serum albumin proteins^20^ and thermochromic ink,^5^ which exhibit progressive color change and irreversible MR changes upon heating at ablative temperatures. Although visualization of lesions is a substantial advantage of this phantom category, permanent changes make them unsuitable for repeated use. In addition, the ingredients of PAA‐based gels are considered to have toxic environmental effects restricting their wider utilization.^2^

On the other hand, agar gels serve as a more natural alternative having easier and more cost‐effective preparation and storage.^3^ They can be easily shaped to any configuration to form phantoms of durable stability. Their tissue‐like MR signal makes them the material of choice for MRI studies.^21^, ^22^, ^23^, ^24^, ^25^, ^26^, ^27^ In fact, a wide variety of agar‐based phantoms simulating specific body parts, such as prostate,^27^ carotid,^21^ and brain,^26^ have been proposed in the literature for evaluating new MR protocols and imaging techniques. This phantom type has also been quite widely used for thermal studies with FUS,^3^, ^28^, ^29^, ^30^, ^31^ where agar served as the gelling agent, and proper concentration of other materials was added to modify mainly the thermal and acoustical properties depending on the tissue to be mimicked. Notably, quite large data on the acoustical properties of agar phantoms exist in the literature. Silicon dioxide,^28^ graphite, and cellulose particles,^32^ are examples of ingredients that served as attenuation modifiers enhancing ultrasonic scattering. Accordingly, evaporated milk was shown to be a prominent absorber of acoustic energy, also enhancing ultrasonic attenuation,^4^ whereas ultrasonic velocity can be adjusted by incorporating proper concentration of glycerol.^32^

Although more limited research has been applied in the investigation of MR parameters of agar‐based phantoms, some interesting trends become apparent through the literature. Agar turned out to be the prominent *T*2 modifier even in the case where another material serves as the gelling agent.^25^, ^33^ *T*1 was predominantly tailored by varying the concentration of paramagnetic ion salts^22^, ^23^ and copper ions.^25^

We have previously proposed and characterized several agar‐based phantoms by estimating critical tissue properties, including the mass density, speed of sound, acoustic attenuation, acoustic impedance, thermal diffusivity, specific heat, and thermal conductivity.^3^, ^14^, ^29^ Given the current need for TMPs that can also replicate critical MR parameters, as well as the lack of targeted research on trends between added ingredients and resultant MR properties of agar phantoms, we have investigated the MR relaxation times of different mixtures of agar‐based phantoms previously proposed by our group.^3^, ^14^

## 2 METHODS

This study concerns the development and MR characterization of agar‐based phantoms. No animals or patients were involved in the study. Therefore, no informed consent from patients or approval from an ethics committee was required.

### 2.1 Phantoms' development

**FIGURE 1:**
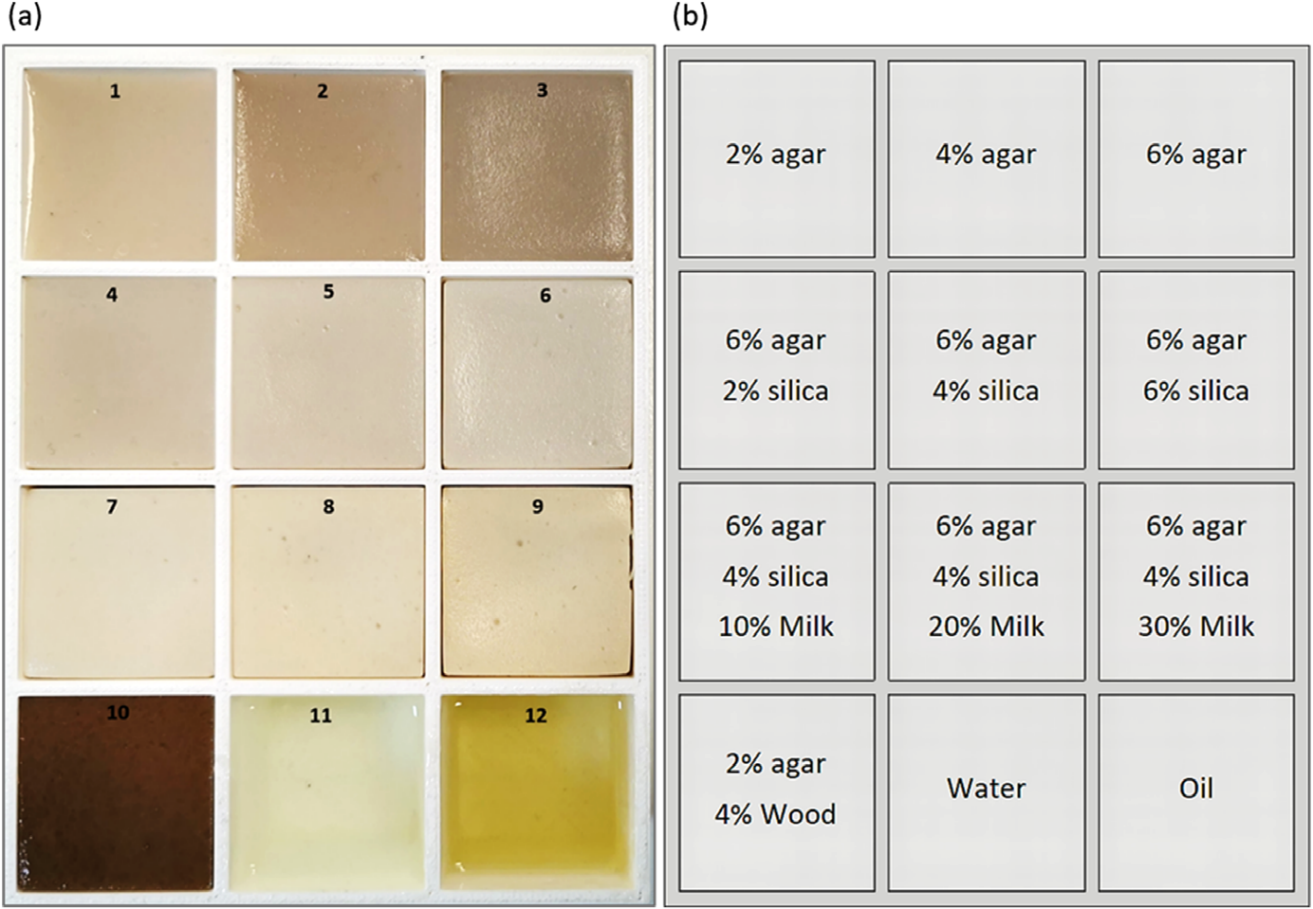
(a) Photo of the phantoms in the container and (b) the corresponding recipe used for each one

Ten agar‐based phantoms with different concentrations of additives were prepared and contained in a rectangular container. The container was specially developed having 12 compartments to accommodate the TMPs and two reference liquids (water and oil), as shown in Figure 1a. Figure 1b shows the composition of the corresponding materials used in each insert. The preparation process of the phantoms was previously described in detail.^3^

Three phantoms with varying agarose (Merck KGaA, EMD Millipore Corporation, Darmstadt, Germany) concentrations of 2%–6% weight per volume (w/v) were prepared to assess the role of agar as a modifier of the relaxation times. The effect of varying silicon dioxide (Sigma‐Aldrich, St. Louis, Missouri, United States) concentration (2%–6% w/v) on the relaxation times was then investigated using a certain amount of 6% w/v agar. Finally, various amounts of evaporated milk (Nounou, Friesland Campina, Marousi, Greece) were added in phantoms with fixed concentrations of 6% w/v agar and 4% w/v silicon dioxide. The volume per volume (v/v) concentration of evaporated milk varied from 10% to 30%.

Agar‐based phantoms doped with wood powder were previously found to possess lower thermal conductivity compared to the silica/evaporated milk doped phantoms and an acoustic absorption coefficient closer to that of soft tissue.^3^ Thereby, another phantom containing 2% w/v agar and 4% w/v wood powder was constructed according to the procedure previously described by our group.^3^

### 2.2 MR properties of phantoms

#### 2.2.1 Physical principle of MR relaxation times

Tissues are characterized by two relaxation times, which describe the rate at which protons return to equilibrium following a radiofrequency pulse. The maximum transverse magnetization $M_{0xy}\;$after a radiofrequency pulse is lost with time as the spinning protons interact with each other and lose phase coherence. T2 is the transverse relaxation time, which by default equals the time needed for the transverse magnetization $(M_{xy})\;$to fall to approximately 37% of its maximum value ($M_{0xy}$) and mathematically is defined by the following equation^34^:

(1) $$M_{xy}=M_{0xy}\;e^{-\frac{\mathrm{TE}}{T2}}$$

where TE is the echo time.

Accordingly, T1relates to the realignment of spinning protons with the external magnetic field and is defined as the time required for the longitudinal magnetization $\left(M_{z}\right)$ to recover to approximately 63% of its maximum value $\left(M_{\mathrm{oz}}\right)$. Mathematically, this recovery is described as follows^34^:

(2) $$M_{z}=M_{0z}\;\left(1-2e^{-\frac{\mathrm{TI}}{T1}}\right)$$

where TI represents the inversion time. It is noted that this expression assumes that the repetition time (TR) is sufficiently longer than the T1to be estimated.

#### 2.2.2 Estimation of MR relaxation parameters

**FIGURE 2:**
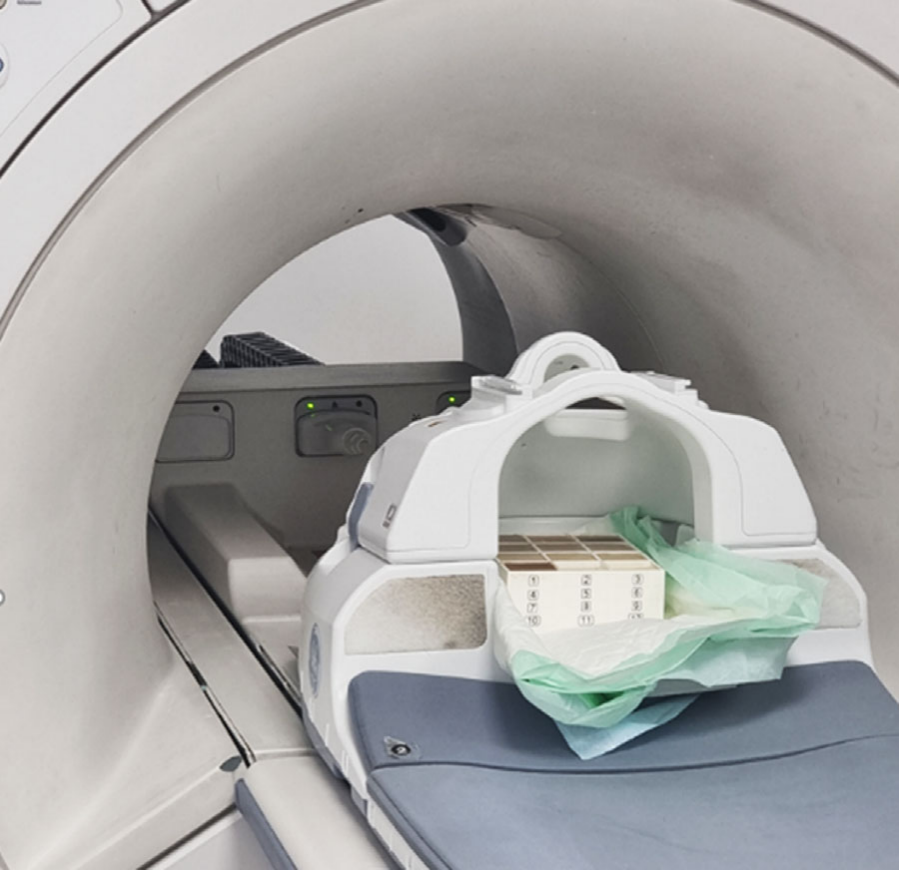
The phantom container positioned on the magnetic resonance imaging (MRI) table within the posterior head and face part of the head/neck/spine (HNS) coil

The developed phantoms were imaged in a 1.5 T MRI scanner (GE Signa HD16; GE Healthcare, Milwaukee, Wisconsin, USA) to demonstrate the effect of the various additives on their MR properties. The container was covered by the posterior head and face part of a head/neck/spine coil (Signa 1.5T, 16 channel, GE Healthcare) as shown in Figure 2.

A 2D MultiEcho imaging sequence was used for assessing the transverse relaxation time. Multiple coronal scans were obtained at variable TEvalues, thus demonstrating the transverse magnetization exponential decay. T2 was estimated by fitting the measured signal intensity (SI) over TE to the exponential function of Equation (1). The images were acquired with the following parameters: TR = 200 ms, TE = 12.0–250.0 ms, flip angle = 90°, echo train length (ETL) = 4, pixel bandwidth (pBW) = 122.1 kHz, matrix size = 160 × 128, field of view = 260 × 260 mm^2^, slice thickness = 7 mm, and number of excitations (NEX) = 0.75.

Accordingly, *T*1‐weighted (*T*1W) inversion recovery (IR) fast spin echo (FSE) images of the phantoms were obtained at variable TIs for *T*1 mapping. The data were fitted into Equation (2) to estimate the longitudinal relaxation time (*T*1). Two‐dimensional axial images were acquired with the following parameters: TR = 7000 ms, TE = 9.94 ms, TI = 50 ‐ 3000 ms, flip angle = 90°, ETL = 9, pBW = 27.10 kHz, matrix size = 192 × 128, field of view = 260 × 260 mm^2^, slice thickness = 7 mm, and NEX = 1.

The methodology for estimating the MR relaxation times of each phantom included both region of interest (ROI) and voxel‐by‐voxel analysis. The ROI approach for *T*1 and *T*2 mapping involved measurement of the SI in specific predefined ROI in the phantom for each TI and TE, respectively. The mean values of the SI were fitted to Equations (1) and (2). Similarly, in the voxel‐based approach, parametric maps were derived from the series of images by fitting the mathematic models to the acquired data for each individual voxel through automated algorithmic processing.

## 3 RESULTS

**FIGURE 3:**
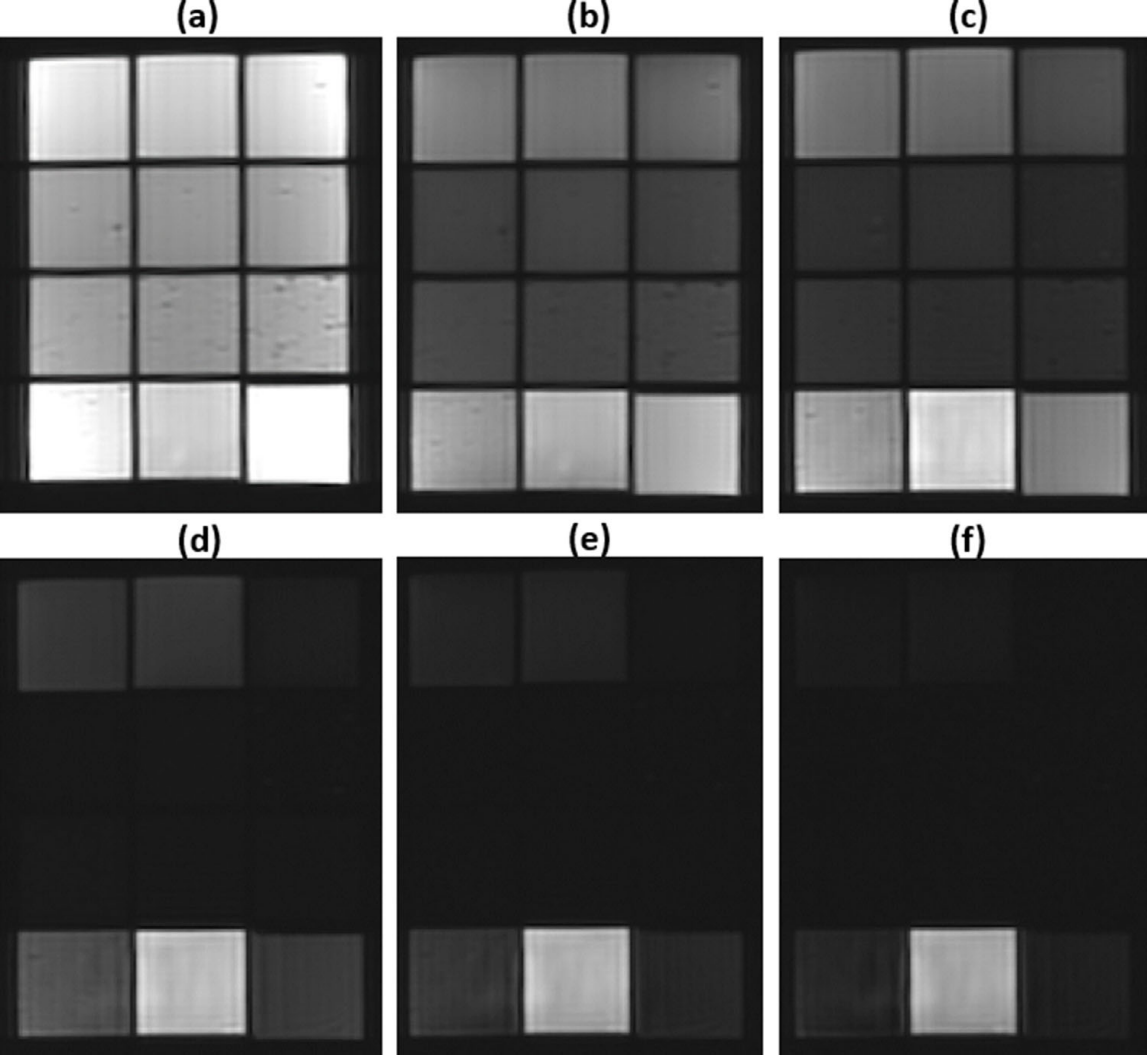
Coronal slices acquired using a 2D multi‐echo sequence at echo times of (a) 12 ms, (b) 36 ms, (c) 50 ms, (d) 100 ms, (e) 150 ms, and (f) 200 ms

**FIGURE 4:**
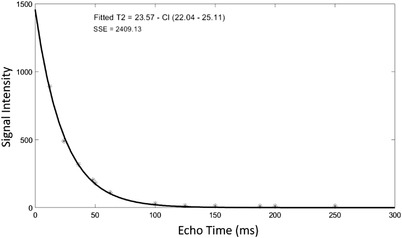
Plot of the mean signal intensity measured from the multi‐echo images using the region of interest approach against echo time for phantom 7 (6% w/v agar, 4% w/v silica, and 10% v/v milk). SSE corresponds to the sum of square errors. CI corresponds to 95% confidence intervals for the estimated values

**FIGURE 5:**
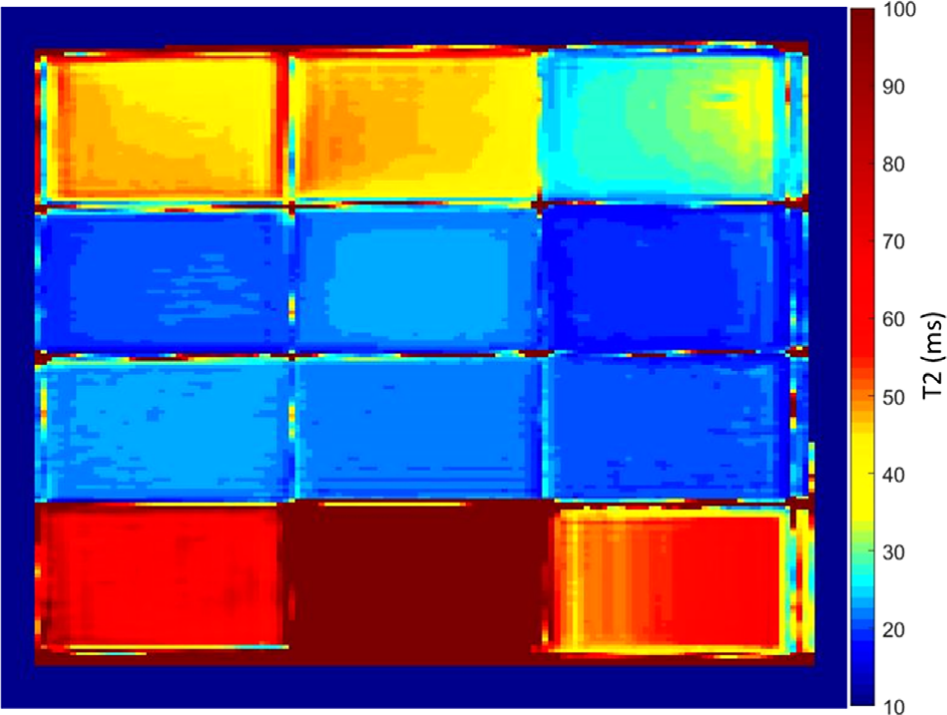
*T*2 parametric map of phantoms. The map was generated by voxel‐based analysis of a series of 2D coronal MultiEcho images with different echo time values (12–250 ms)

The phantoms were initially scanned in the coronal plane using a multi‐echo sequence with TE values ranging from 12 to 250 ms. Figure 3 shows indicative MR images acquired at various TEs within this range. Figure 4 shows an indicative graph of the estimated mean SI in a predefined ROI of the phantom in insert 7 (6% w/v agar, 4% w/v silica, and 10% v/v milk) plotted against the TE, demonstrating the rate of transverse magnetization decay. The *T*2 parametric map of the phantoms as generated by the voxel‐by‐voxel analysis is presented in Figure 5.

**FIGURE 6:**
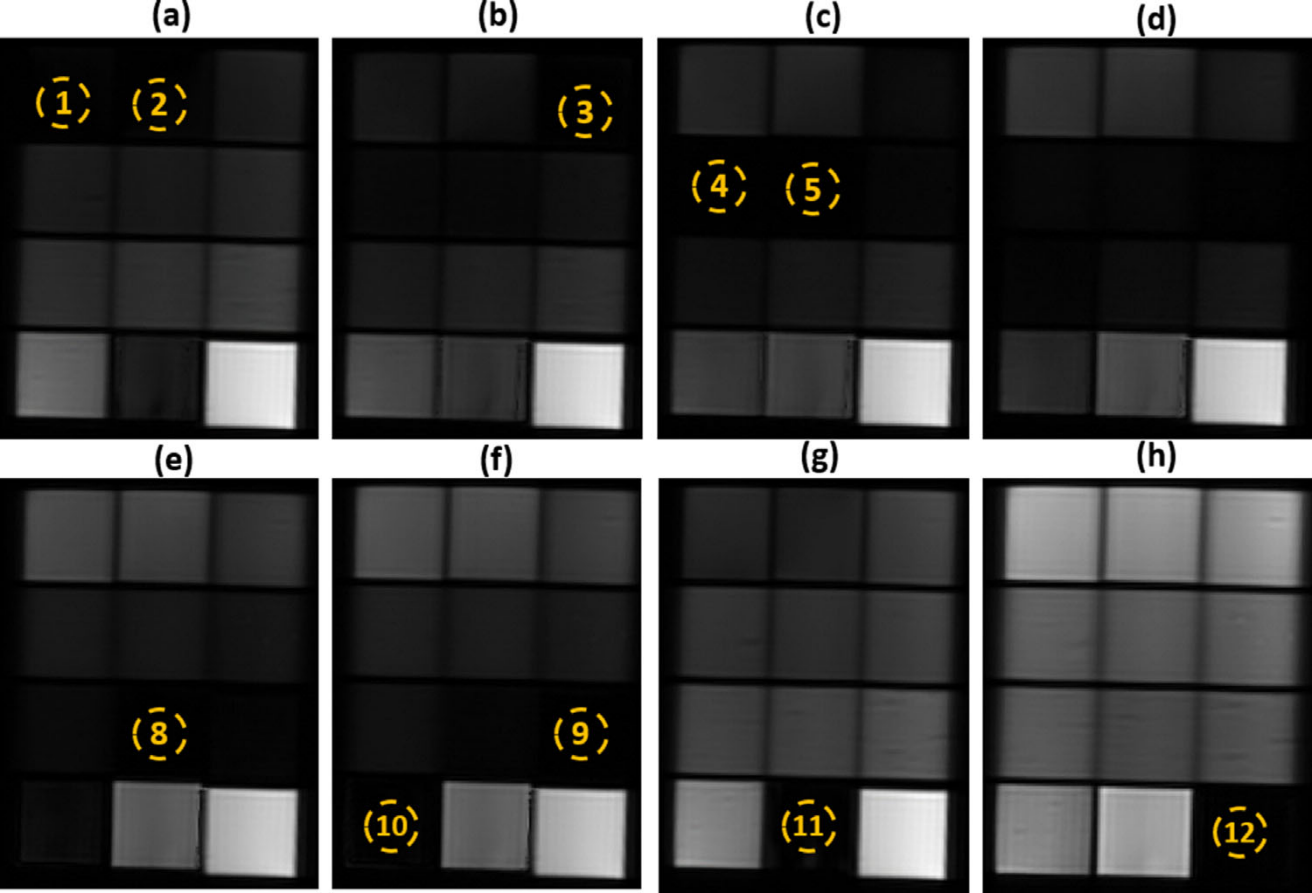
Axial slices of the phantoms acquired using a 2D *T*1W IR FSE sequence at inversion times of (a) 1200 ms, (b) 1000 ms, (c) 900 ms, (d) 800 ms, (e) 650 ms, (f) 600 ms, (g) 1500 ms and (h) 125 ms. The material shown in the yellow dotted circle has the lowest SI

**FIGURE 7:**
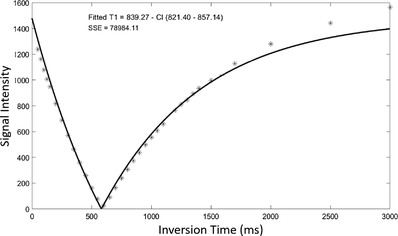
Plot of the mean signal intensity measured from the *T*1‐weighted inversion recovery fast spin echo (*T*1W IR FSE) images using the region of interest approach against inversion time for phantom 9 (6% w/v agar, 4% w/v silica, and 30% v/v evaporated milk). SSE corresponds to the sum of square errors. CI corresponds to 95% confidence intervals for the estimated values

**FIGURE 8:**
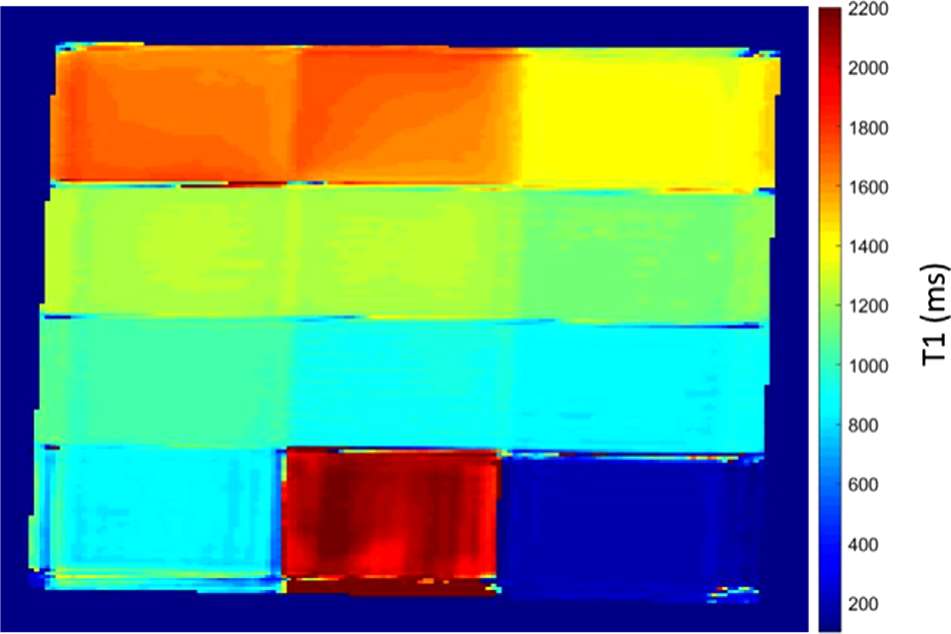
*T*1 parametric map of phantoms. The map was generated by voxel‐based analysis of a series of 2D axial *T*1‐weighted inversion recovery fast spin echo (*T*1W IR FSE) images with different inversion time values (50–3000 ms)

Imaging of phantoms was then done in the axial plane using a *T*1W IR FSE sequence at various TI values in the range of 50 to 3000 ms. Indicative results are presented in Figure 6, where the yellow dotted circles indicate the phantoms with the lowest SI for each TI. A typical graph of the change in SI with increasing TI value as estimated by the ROI approach is shown in Figure 7, which demonstrates the mean SI versus TI for the phantom in insert 9 (6 % w/v agar, 4 % w/v silica, and 30 % v/v evaporated milk). The *T*1 parametric map generated by the voxel‐by‐voxel analysis is presented in Figure 8.

**FIGURE 9:**
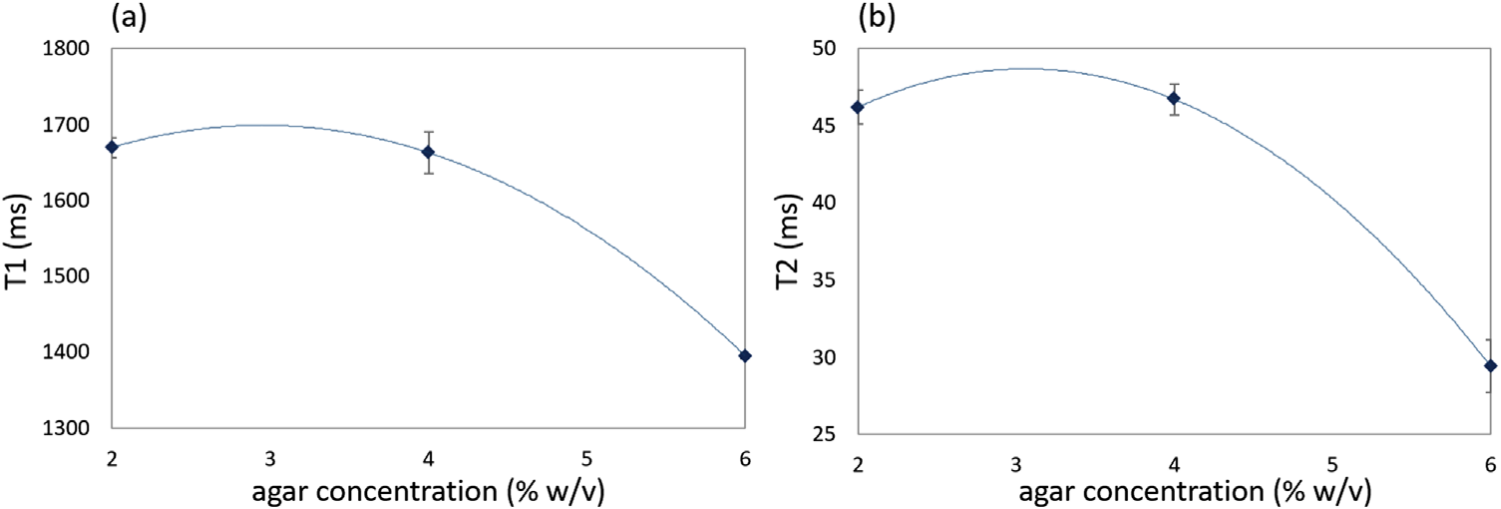
The mean (a) *T*1 and (b) *T*2 values plotted against the agar concentration. The data points are fitted by polynomial regression where the error bars correspond to the standard deviation as estimated by voxel‐based analysis

**FIGURE 10:**
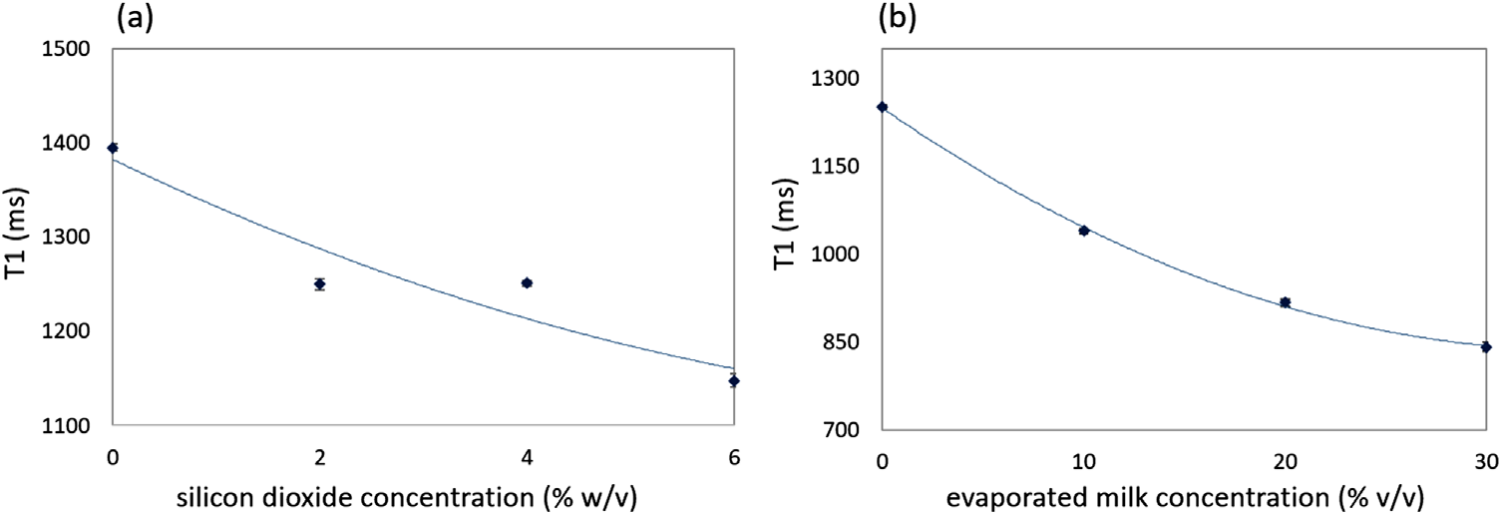
The mean *T*1 value plotted against (a) the silica concentration for a fixed amount of 6% w/v agar and (b) the evaporated milk concentration for fixed amounts of 6% w/v agar and 4% w/v silica. The data points are fitted by polynomial regression where the error bars correspond to the standard deviation as estimated by voxel‐based analysis

The mean value of the *T*1 and *T*2 relaxation times and the corresponding standard deviations for each phantom as estimated by the voxel‐based approach are listed in Table 1. Figure 9A,B shows the estimated *T*1 and *T*2 values plotted against the agar concentration, respectively, where the data points were fitted to a 2nd order polynomial (*R* ^2 ^= 1) using non‐linear least square regression. Accordingly, the effect of varying amounts of silicon dioxide and evaporated milk on *T*1 is presented in Figure 10, in which the graphs also represent 2nd order polynomials (*R* ^2 ^= 0.899 and  0.999, respectively).

**TABLE 1:** Mean *T*1 and *T*2 and standard deviation (SD) of phantoms as estimated by voxel‐based analysis

**Phantom #**	**Recipe**	***T*2 (ms)**	**SD (ms)**	***T*1 (ms)**	**SD (ms)**
1	2% agar	46.2	1.1	1669.5	13.3
2	4% agar	46.7	1.0	1662.7	27.6
3	6% agar	29.4	1.7	1394.9	3.8
4	6% agar, 2% silica	20.9	0.4	1249.8	6.4
5	6% agar, 4% silica	23.4	0.2	1251	3.0
6	6% agar, 6% silica	19.0	0.3	1147.7	7.3
7	6% agar, 4% silica, 10% milk	23.0	0.2	1038.8	4.7
8	6% agar, 4% silica, 20% milk	21.8	0.2	916.8	6.4
9	6% agar, 4% silica, 30% milk	20.1	0.23	841.3	8.1
10	2% agar, 4% wood	65.2	2.7	837.5	12.0
11	Water	–	–	2125.6	42.1
12	Oil	55.2	3.4	193.3	1.8

## 4 DISCUSSION

Ten different agar‐based TMPs were prepared and imaged in a 1.5 T MRI scanner to assess their suitability to match the MR properties of real tissue. It is widely known that the MR SI depends on the characteristic relaxation times of the imaged object.^16^ A typical methodology that makes use of this dependency was followed for *T*1 and *T*2 mapping. A series of MultiEcho images were acquired at different TE values for *T*2 mapping. Accordingly, *T*1 mapping was performed by acquiring *T*1W IR images at different TIs after applying the inversion pulse (180°). The relaxation times were estimated by fitting the acquired data to the signal decay and recovery curves, respectively, through both ROI and voxel‐based approaches.

Pure agarose phantoms were initially scanned to demonstrate the effect of agar concentration on the relaxation times. Both relaxation parameters showed similar behavior. Increment of the agar concentration from 2% to 4% w/v had no impact on the resultant relaxation times, whereas both *T*1 and *T*2 showed a noticeable decrease as agar concentration increased to 6% w/v. It is notable that the relation between both relaxation times and the agar concentration can be perfectly modeled as a 2nd‐degree polynomial function (*R* ^2 ^= 1). Although the present results are in line with previous studies^25^, ^33^ proposing agarose as a *T*2 modifier, they suggest that this only applies for agar concentrations of 4% w/v and above.

The change in MR properties of agar gels upon the addition of various amounts of silicon dioxide and evaporated milk was then assessed. The addition of silica particles further lowered the relaxation times. However, no specific trend became apparent with increasing silicon dioxide concentration for none of the relaxation times. The results further suggest that the addition of evaporated milk has no specific impact on *T*2, whereas a noticeable decrease is observed in the case of the longitudinal relaxation time (*T*1). In fact, the *T*2 relaxation time of milk‐doped agar gels (6% w/v agar and 4% w/v silicon dioxide) remained similar to those containing only silicon dioxide. On the contrary, milk‐doped agar/silica gels exhibit noticeably shorter *T*1 relaxation times, with increasing evaporated milk concentration (0%–30 % v/v) resulting in a gradual reduction of *T*1 in a 2nd order polynomial manner (*R* ^2 ^= 0.999). This implies that *T*1 and *T*2 may be changed independently; however, this should be further investigated. It is also noted that milk concentrations higher than 30%, which would probably lower *T*1 even more, were not attempted because they would result in loose phantoms.^35^

Our results further demonstrated that agarose could also serve as a *T*1 modifier. However, it seems that *T*2 depends more strongly on the amount of agarose and is not remarkably affected by the concentration of other additives. Note that with increasing agar concentration at TEs of 36–200 ms the signal drops (Figure 3). On the contrary, with a fixed agar concentration of 6% w/v and increasing silica, the signal does not change much. Note also that the same holds by increasing the milk concentration. This result ties well with previous studies wherein *T*2 was mostly defined by the gelling agent concentration, whereas *T*1 was mainly varied by incorporating different concentrations of paramagnetic ion salts.^22^, ^23^, ^24^

The MR parameters of TMPs have been previously shown to be dependent on the concentration of scatterers.^4^, ^36^ In a study by Hofstetter et al.,^4^ a decrease of *T*2 occurred with increasing concentration of psyllium husk in gelatin‐based phantoms. A similar trend was reported in a study by Huber et al.,^36^ wherein the inclusion of glass beads shortened *T*1 of an agar/gelatin‐based phantom. Herein, the addition of wood scatterers also lowered *T*1 of pure agar gel (2% w/v). The phantoms doped with silicon dioxide appeared with lower relaxation times compared to agar only gels as well. However, it should be emphasized that the trend with increasing silica is not reliable as the distribution of silica in the material might be random.

Overall, the MR relaxation times of the proposed agar‐based phantoms are comparable with the values reported for body tissues. A review article by Bottomley et al.^37^ reports *T*2 relaxation times of soft tissues ranging roughly between 40 and 80 ms. Herein, the estimated *T*2 values ranged from a minimum value of 19.0 (±0.3) ms for the phantom in insert 6 (6% agar, 6% silica) to a maximum value of 65.2 (±2.7) ms for the phantom in insert 10 (2% agar and 4% wood). Authors also report a mean *T*2 in adipose tissue of 84 (±36) ms,^37^ which compares well with the value of 55.2 (±3.4) ms found by the current study for oil. At this point, it should be noted that the *T*2 measurement of water is not reported because of insufficiently high echo times due to machine limitations. Regarding the longitudinal relaxation time, the estimated *T*1 values range from 837.5 (±12) ms to 1669.5 (±13.3) ms for the phantoms in inserts 10 and 1, respectively. These estimates are partly consistent with the literature documenting *T*1 values for soft tissues harshly between 500 and 1000 ms.^38^

The several phantom recipes can be matched with specific tissue types through a more detailed comparison with the cited literature. For instance, by using concentrations of 2% w/v agar and 4% w/v wood (phantom in insert 10), a *T*2 value of 65.2 (±2.7) ms was found, which agrees with the value of 61 (±11) ms reported by prior research for the kidney tissue.^38^ Regarding the *T*1 relaxation time, the value of 837.5 (±12.0) ms estimated by the current study is quite higher than the value of 709 (±60) reported literally for the kidney.^38^ Accordingly, the silica/milk doped phantom in insert 7 was found to possess MR properties close to that of skeletal muscle and heart tissue (at 1.5 T).^38^ Note that the high *T*1 values estimated for the agar only phantoms can only be well correlated to the *T*1 relaxation times of human blood.^38^

Finally, it is important to notify the reader that quantitative relaxation times are particularly dependent upon the used pulse sequence.^39^, ^40^ Furthermore, although the proposed multi‐echo SE sequence is conventionally selected for *T*2 relaxometry significantly reducing the scan time of single‐echo sequences, it is accompanied by the limitation that *T*2 overestimation may occur when the applied 180°C RF pulses fail to perfectly refocus magnetization, which is actually challenging in real practice.^41^ Accordingly, *T*1 values may also be underestimated if the TR values employed in the IR sequence are not chosen properly.^40^ Therefore, optimal imaging parameters should be employed for accurate and reliable *T*1 and *T*2 determination. It is thus recommended that the variance in relaxation values between different sequences, as well as their dependence on precise pre‐scan settings should be taken into consideration when comparing the current results with those of other similar studies.^42^

## 5 CONCLUSIONS

Overall, the proposed phantoms can be formed in any configuration while maintaining the desired mechanical strength upon solidification. Their manufacturing process is easy, and the materials used are cheap and easy to obtain. The current findings suggest that the transverse relaxation time (*T*2) of agar‐based phantoms can be predominantly tailored by varying the agar concentration. The inclusion of silicon dioxide lowers both relaxation times, whereas increasing evaporated milk concentration results in a gradual reduction of the longitudinal time (*T*1). Accordingly, the *T*1 and *T*2 relaxation parameters of several body tissues can be accurately matched by a proper concentration of these inclusions. Therefore, the proposed phantoms have great potential for use with the continuously emerging MRgFUS technology, also given their previously demonstrated feasibility to emulate all critical thermal and acoustical properties of human tissues.

## CONFLICT OF INTEREST

The authors declare that they have no conflict of interest.

## AUTHOR CONTRIBUTIONS

Anastasia Antoniou contributed to analyzing the findings and drafting the work. Leonidas Georgiou contributed to the analysis and interpretation of MR data for the work. Theodora Christodoulou contributed to the acquisition of MR data for the work. Natalie Panayiotou contributed to the acquisition of MR data for work. Cleanthis Ioannides contributed to the MRI experiments and interpretation of results. Nikolaos Zamboglou contributed to the MRI experiments and interpretation of results. Christakis Damianou served as the scientific coordinator and supervised the development of phantoms, implementation of the experiments, and drafting of the manuscript.

## Data Availability

The data that support the findings of this study are available from the corresponding author upon reasonable request.
